# Exercise-referral scheme to promote physical activity among hypertensive patients: design of a cluster randomized trial in the Primary Health Care Units of Mexico’s Social Security System

**DOI:** 10.1186/1471-2458-14-706

**Published:** 2014-07-09

**Authors:** Katia Gallegos-Carrillo, Carmen García-Peña, Jorge Salmerón, Velia Nelly Salgado-de-Snyder, Gabriel Vázquez-Cabrer, Felipe Lobelo

**Affiliations:** 1 Unidad de Investigación Epidemiológica y en Servicios de Salud, Instituto Mexicano del Seguro Social, Boulevard Benito Juárez No. 31. Centro. C.P. 62000, Cuernavaca, Morelos, México; 2 Unidad de Investigación Epidemiológica y en Servicios de Salud. Área Envejecimiento, Instituto Mexicano del Seguro Social, México, D.F. México; 3 Centro de Investigación en Sistemas de Salud, Instituto Nacional de Salud Pública, Cuernavaca, Morelos, México; 4 Unidad de Rehabilitación Cardíaca, Hospital Ángeles de las Lomas, Huixquilucan, Estado de México, México; 5 Exercise is Medicine Global Research Center, American College of Sports Medicine, Indianapolis, Indiana, USA; 6 Centro de Investigación en Salud Poblacional, Instituto Nacional de Salud Pública, Cuernavaca, Morelos, México

**Keywords:** Exercise-referral, Physical activity, Hypertension, Mexico

## Abstract

**Background:**

Although the benefits of physical activity (PA) on to prevent and manage non-communicable diseases are well known, strategies to help increase the levels of PA among different populations are limited. Exercise-referral schemes have emerged as one effective approach to promote PA; however, there is uncertainty about the feasibility and effectiveness of these schemes in settings outside high-income countries. This study will examine the effectiveness of a scheme to refer hypertensive patients identified in Primary Health Care facilities (PHCU) of the Mexican social security institution to a group PA program offered in the same institution.

**Methods and design:**

We will describe the methods of a cluster randomized trial study designed to evaluate the effectiveness of an exercise referral scheme to increasing physical activity in hypertensive patients compared to a non-referral scheme. Four PHCU were selected for the study; the PHCU will take part as the unit of randomization and sedentary hypertensive patients as the unit of assessment. 2 PHCU of control group (GC) will provide information to hypertensive patients about physical activity benefits and ways to increase it safely. 2 PHCU of intervention group (IG) will refer patients to sports facilities at the same institution, to follow a group-based PA program developed to increase the PA levels with a designed based on the Transtheoretical Model and Social Cognitive Theory. To evaluate the effects of the intervention as well as short-term maintenance of the intervention’s effects, PA will be assessed at baseline, at 24 and 32 weeks of follow-up.

The main outcome will be the difference before and after intervention in the percentage of participants meeting recommended levels of PA between and within intervention and control groups. PA will be measured through self-report and with objective measure by accelerometer.

**Discussion:**

This study will allow us to evaluate a multidisciplinary effort to link the primary care and community-based areas of the same health care system. Our findings will provide important information about the feasibility and effectiveness of an exercise-referral scheme and will be useful for decision-making about the implementation of strategies for increasing PA among hypertensive and other clinical populations in Mexico and Latin America.

**Trial registration:**

Clinicaltrials.gov Identifier: NCT01910935. Date of registration: 07/05/2013.

## Background

The benefits of physical activity (PA) on health have been well documented [1-3]. However, a large proportion of the world population fails to meet current global physical activity recommendations (at least 150 minutes of moderate-to-vigorous PA/week) [4]. In 2010 the global prevalence of inactivity was estimated at 32%, and as much as 40% of the population in the Americas [5]. In Mexico, the prevalence of physical inactivity has increased 6%, from 13.4% to 19.4% in only six years [6].

Globally, the magnitude of the physical inactivity problem is worse in poorer regions of the world. For example, until 80% of mortality due to NCD’s and physical inactivity occurs in lower-middle income countries.^7^ As part of the strategies developed to prevent and control NCDs, Mexico’s Social Security Institution (MSSI), which covers 49.2% of the Mexican population, is providing comprehensive health care and referrals for PA programs for patients [7]. However, 16.8% are physical inactive and in a whole 83% of do not meet PA recommendations [8].

Increasing physical activity to recommended levels would help eliminate 6% to 10% of the major Non-Communicable Diseases (NCDs), including coronary heart disease (CHD), type 2 diabetes, breast and colon cancers [9]. Physical inactivity contributes to and aggravates NCDs like hypertension (HTN), [10] which constitutes the leading cause of NCD visits at the MSSI, [11] and the proportion of newly diagnosed MSSI patients with HTN has increased by 30% in only five years [12]. Complications associated with HTN consume between 6 and 8% of the total health care expenditure of the Mexican health system [11,13]. Adding physical activity to the HTN medical treatment paradigm for HTN can help control blood pressure, reduce mortality and complications and help curb the health care [14].

The Primary Health Care (PHC) setting has been identified as a base for PA-promotion strategies since it is a point of contact with hypertensive patients, and since basing interventions there would facilitate the continuity of care and integration of multiple disciplines into treatment [15]. PHC physicians have been seen as source of health promotion and powerful agents for physical activity promotion [16]. However, through the years strategies focusing exclusively on physician involvement have had little success. Insufficient health promotion training, lack of time of medical consultation, and lack of physician confidence that their recommendations for healthy lifestyles are useful for patients, has been the aim barriers to achieve it [17-19]. Therefore, it appears that PA counseling and PHC-based PA-referral schemes as an inclusive strategy utilizing available staff and facility resources to help people to become physically active might function better in this settings, since both have proven effective in helping inactive people to meet PA recommendations [20-22].

Systematic reviews of exercise-referral schemes demonstrate that challenges remain in terms of increasing patients’ acceptance of and adherence to PA programs [19-23]. However the results of these studies are from high-income countries, and little is known about effective ways to increase PA in countries with different income levels. These lower income countries are the focus of only 10% of the global research but house 90% of the world’s population [24]. If lifestyle changes such as increasing PA practice are to be implemented worldwide, it is essential to understand the mechanisms underlying the behavior of diverse populations.

In the context of studies about effectiveness of PA-referral schemes, to date there is lack of proven evidence of its effectiveness with objective outcome measures of PA [25], since interventions’ effects have been documented exclusively by self-report [21,26]. Therefore, this study will estimate the effectiveness of PA referral scheme with an objective PA assessment. This will be the first study of PA-referral scheme for chronically ill patients in the Mexican Health System.

The aim of this study is to evaluate the effectiveness of an MSSI PHC-based physician to a PA- referral scheme designed to help chronically ill patients to increase PA, in order to perform recommended levels of PA (150 minutes at week of moderate to vigorous intensity).

## Methods and design

### Setting

The Mexican Social Security Institute (MSSI) includes offices for primary, secondary and tertiary health services as well as facilities designed to develop PA programs (sports fields, gyms, pools and outdoor spaces for PA) called Social Security Centers (SCC). The SCC have specialized staff to guide and assist on promotion and practice PA in all potential participants.

### Design and recruitment process

This is a cluster randomized trial, with the primary health care unit (PHCU) as the unit of randomization and hypertensive patients as the unit of assessment, to evaluate the impact of a PA-referral scheme to help patients increase their PA levels and meet recommended PA guidelines. Four MSSI PHCU facilities will be assigned in a randomized way to intervention (2 PHCUs) and control groups (2 PHCUs). In all PHCUs, primary care physicians will identify hypertensive patients based on the following criteria: women and men with MSSI affiliation, between 35 and 70 years old, less than 5 years from hypertension diagnosis and/or without pharmacological treatment self-reporting willingness to engage in a PA program but currently not meeting PA recommendations (< 150 minutes per week of moderate to vigorous intensity PA). Using a standardized protocol, physicians will quickly screen for these criteria and will refer eligible patients to the study suite, located in the same PHCU facility. Trained staff will verify that potential participants meet the inclusion criteria described below, to confirm that they can safely engage in the PA program.

Inclusion criteria:

a) Adults between 35 and 70 years old; b) both sexes; c) patients with hypertension diagnosis within five years or less and/or without pharmacological therapy for hypertension (according with JNC 7 criteria) [27]; d) Not meeting PA recommendations (<150 minutes at week of moderate or vigorous intensity), as measured with the short-form of the International Physical Activity Questionnaire (IPAQ) [28]; e) currently in the contemplation or preparation stage for PA, according to Transtheoretical model of behavioural change [29]; f) low to moderate level of cardiovascular risk, according to the Guidelines for exercise testing and recommendations of the American College of Sports Medicine (ACSM) [30]. A pre-participation screening questionnaire from American College of Sports Medicine (ACSM) and ACSM [31], will be used to evaluate health status, medical history of cardiovascular, metabolic or lung disease, signs or symptoms suggestive of these conditions and number of cardiovascular risk factors. Symptoms that require a complex evaluation will be confirmed by medical staff; g) blood pressure levels: systolic blood pressure levels ≤ 160 mm Hg or diastolic ≤ 100 mm Hg [32,33]; [h) Body Mass Index < 35; i) biochemical markers in the following ranges: total cholesterol ≤ 240 mg/dL, fasting glucose level ≤ 126 mg/dL, triglycerides ≤ 150 mg/dL; j) physical ability to participate in the PA program (without problems that may impede walking or other moderate intensity PA).

**Figure 1:**
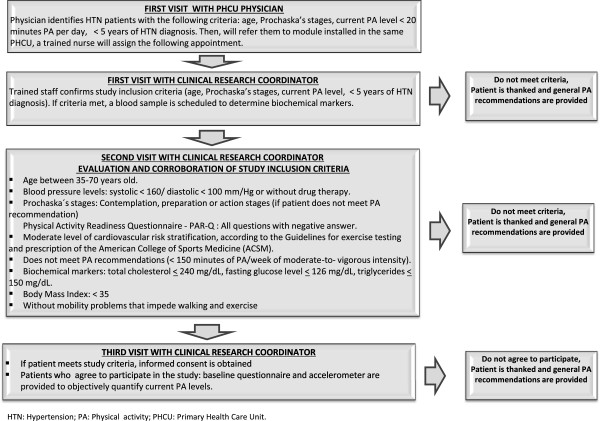
Recruitment flowchart.

Exclusion criteria:

For both the control and intervention groups: currently reporting high levels of PA (> 300 minutes at week of moderate to vigorous intensity) [34], having previously attended a PA program at MSSI facilities. Criteria during PA sessions in the intervention group: systolic/diastolic blood pressure prior to PA sessions > 200/100 mm Hg or during the session > 250/115 mm Hg. The elimination criteria proposed are: less than 80% PA program attendance with or without incomplete information in one of the three data-collection points in the study.The study’s recruitment process is schematized in Figure 1. Patients who meet the criteria previously described will be invited to participate. Patients accepting to participate will provide informed consent and will be formally enrolled.

### Randomization

Randomization will be performed before other study activities, and conducted at MSSI facilities outside the PHCUs to avoid contamination. The 4 PHCUs were randomized into the intervention group (IG; 2 centers) and control group (CG; 2 centers) using sealed envelopes by a health researcher who was not involved in this study. To avoid contamination, that PHCUs selected, are on average 5.5 kilometers (kms) from the place where patients will be referred (IG at 5.1 kms. and CG at 6.6 kms.), within the urban area of the city and public transportation available, average transfer time is 20 to 30 minutes.

#### Baseline assessment

**Figure 2:**
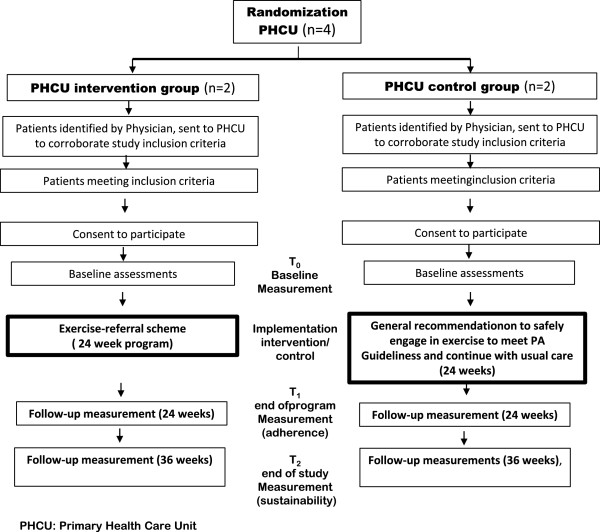
Study design flowchart.

During this assessment a questionnaire will be administered and functional capacity measurements will be taken. To evaluate the effects of the intervention, measurements will be taken at baseline, at 24 weeks of study entry baseline, to assess adherence to the intervention, and at 32 weeks of study entry, to determine short-term maintenance of the intervention’s effects. Details of study’s design and flow chart are showed in Figure 2. A trained nurse, part of the research team, will carry out this assessment, duration between 40 to 60 minutes.

### Control group (CG)

Participants in the control group will be informed about health benefits of PA and a balanced diet, and how to safely increase PA levels. Patients in this group will continue receiving their usual care at PHCU facilities. The control group will be invited to undergo two subsequent PA assessments at the PHCU facilities at 24 and 32 weeks of follow-up. The research team will keep in contact with these patients during monthly doctor’s appointment or through a monthly phone call to emphasize the participation in the study and with appointment reminders at weeks 22 and 30.

### Intervention group (IG)

The group-based PA program developed to increase the PA levels of hypertensive patients was designed based on the Transtheoretical Model [29] and Social Cognitive Theory [35,36].

The components of the 24 week PA program are: duration (60 minutes per session), frequency (3 times per week), and intensity (moderate to vigorous). The program will be conducted and supervised by trained staff with experience in handling groups of patients with chronic conditions like diabetes and hypertension; therefore, only typical embodiments of exercise like dancing, supervised walking and swimming will be included.

### Implementation

Patients in the IG, will be invited for following activities during the first three sessions of the PA program at the SCC:

1) Select and enroll in their PA group. Each group will start with a minimum of 5 patients and a maximum of 20. Therefore, between 6 and 8 new groups may open during the intervention, depending on the flow of participants. Patient groups were organized based on personal preferences of exercise and for patients with joint or bone problems, swimming will be recommended.

2) During the first session, MSSI staff trained in guiding the PA program will administer the functional capacity test, to personalize the PA prescription and determine the PA intensity each patient will be instructed to exercise at the start of the program. PA intensity will be increased gradually, depending on patient characteristics and training progression for each patient. PA prescription will start at 55% of the patient’s maximum heart rate (MHR), and gradually increase to 75% of MHR to enhance safety and efficacy. The trained staff will instruct patients how to monitor their heart rate before, during and after PA sessions and to be alert to fluctuations outside the ranges. A general description of the PA program progression follows.

**Level 1 or induction:** between the initial 4 to 6 weeks, during which the patients will adopt a regular exercise habit.

**Level 2 or development:** during weeks 7 to 12 in which there will be a gradual increase in exercise volume (duration and intensity) every 2 weeks.

**Level 3 or maintenance:** beginning at the 13th week, when the patient has started to reach an improved level of functional capacity and exercise tolerance, allowing him or her to maintain the same routine in duration and intensity during group-based exercise classes, while improving free-living and other recreational PA as part of their lifestyle sessions the following weeks.

3) The group-based PA sessions will consist of three stages: warm-up, predominantly aerobic exercise, and recovery. The components of PA program are shown in Table 1. The trained staff will also record participants’ self-reported recreational PA outside the SCC.

**Table 1:** Components of physical activity program

**Physical activity phases**	**Level 1 induction (Week 2-6) duration, target intensity**	**Level 2 development (Week 7-12) duration, intensity**	**Level 3 maintenance (Week 13-23) duration, intensity**
**Warm-up phase:** Involving all parts of the body: neck, upper limbs, lower limbs	10 minutes, 50% of MHR	10 minutes, 50% of MHR	10 minutes, (MHR) 50%
**Aerobic phase:** Walking, running, dancing or bicycling.	20 minutes (THR) 55%	30 minutes (THR) 55-75%	40 minutes (THR) 60-75%
**Recovery phase:** Gradually reduce the intensity. Stretch, bend and rotate body parts starting with the neck and concluding with the ankles, emphasizing deep breathing.	5-10 minutes	5-10 minutes	5-10 minutes

MHR: Maximum heart rate (220-age). THR: Training Heart Rate.

Reference: Fox III, SM Naughton, JP and Haskell, WL. *Physical activity and the prevention of coronary heart disease. Ann Clin Res* 1971; 3:404-432.

### Outcome assessment

#### Primary outcomes

The main outcome will be the difference before and after intervention in the percentage of participants meeting recommended levels of PA (at least 150 minutes at week of moderate to vigorous intensity) [1,2], between and within intervention and control groups. Based on previous experiences [20-23], the PA program (intervention) will be considered effective if it achieves an increase of 20% in the proportion of hypertensive patients in the intervention group defined as active compared to control group, at the end of the intervention (24 weeks) to evaluate adherence and at 32 weeks to assess the sustainability of intervention effects.

PA levels will be assessed at three time points during the study to determine changes in the following: percentage of participants meeting the PA recommendation, minutes of moderate and vigorous PA performed per week and minutes per day of sedentary behavior. In the intervention group, we will also measure change over time in the following tests of functional capacity: walking distance (6 minute walk test) [37], maximum heart rate and 1 minute heart-rate recovery [38]; these measurements will be taken at baseline, 24 and 32 weeks too. These variables will be used as a complementary assessment of participant’s functional capacity and not as the primary outcome of the study.

#### Secondary outcomes

Outcomes assessed in the intervention and control groups at 24 and 32 weeks will be: a) blood pressure levels; b) biochemical markers: triglycerides, total cholesterol and HDL, fasting glucose; c) anthropometric measures: weight, BMI, hip and waist circumference to calculate waist/hip ratio; d) social/psychological measures: attitude towards PA participation based on Prochaska’s stages of change model [29], PA self-efficacy [39] health related quality of life (SF-12) [40], symptoms of depression (CESD, 20-item version) [41] and social support for PA participation.

### Measurements

Several kinds of data will be collected to measure the intervention’s effects. Measurement procedures are described here:

#### Physical activity measurement

Amount of PA will be measured with objective and self-report instruments.

Objective: Participants will be monitored via ActiGraph GT3X accelerometers (ActiGraph LLC, Ft. Walton Beach, Florida) while exercising. This device has an internal time clock and extended memory, and is able to record and store the magnitude of acceleration and deceleration of movements. The recorded data is scored as a “count”, which can be summed in a specific time interval called an “epoch”. In this study, an epoch will equal sixty seconds. Participants will be instructed to wear the device for 7 days during all waking hours, and to remove it only during swimming, bathing or other contact with water. The accelerometers will be mounted on elastic belts and placed at the right hip. For this study the wear time will be valid, if the patient wears the device for 5 days in one week (including at least 1 weekend day) to accumulate at least 600 minutes daily.

Each patient’s accelerometer record will be analyzed in terms of time (in minutes) performing moderate to vigorous PA per day, to calculate the average time spent (in minutes) of PA during the week. Using this data, we will categorize patients as complying or not complying with recommended levels of PA [2], (150 minutes of PA moderate to vigorous intensity at week) at the different measurement points (baseline, 24 and 32 weeks to IG and baseline and 24 weeks to CG) during the study.

Self-report instrument: PA levels during leisure time will be measured with the short version of the International Physical Activity Questionnaire (IPAQ) [28]. The IPAQ will be used to confirm the patients’ PA levels at screening and at the study measurement points, and will be complemented with a physical activity logs that participants will complete every week and deliver to the research team monthly.

### Clinical measurements

#### Biochemical markers

1) Blood pressure levels: will be measured with a digital instrument following criteria established by the JNC 7 [27]; 2) Fasting glucose levels: blood samples will be taken following a fasting period of at least of 8 hours. The serum glucose determination will be made using the enzymatic calorimetric method. All blood biochemistry tests, including total cholesterol, high density lipids (HDL), and triglycerides will be conducted with a Selectra XL (Randox) in accordance with the procedures of the International Federation of Clinical Chemistry and Laboratory Medicine [42].

#### Anthropometric measurements

Will be carry out by trained nurses, following standard procedures: 1) Weight: will be measured with a TANITA electronic scale, with participants wearing minimal clothes and without shoes; 2) Height: will be measured with a conventional stadiometer, with participants standing without shoes; 3) Body Mass Index (BMI): will be calculated by dividing kilograms by height in meters squared. The data obtained will be categorized according to the following criteria: normal (BMI = 18.5-24.9 kg/m^2^), overweight (BMI = 25–29.9 kg/m^2^) and obese (BMI = ≥ 30 kg/m^2^) [43] 4). Waist circumference: measured at the highest point of the iliac crest at the end of expiration, to the nearest measuring tape point of 0.1 cm. The criteria for abdominal obesity will be: men > 100 cm and women > 88 cm [44] 5); Hip circumference: Participants will be standing with feet separated about 20 cm and weight distributed evenly on both feet, at level of the maximum extent of gluteus in a horizontal plane, verifies that the measuring tape covers at same high the perimeter of the body, near to the skin but without compress [45].

### Demographic, health history and lifestyle measurements

At the three measurement points of the study, participants will complete questionnaires asking about sex, age, marital status, highest educational level, household income; their current, past and family history of hypertension, history of other chronic conditions and diseases, current pharmacological treatments, food intake, tobacco and alcohol consumption. In addition, participants’ attitudes toward behavior change based on Prochaska’s stages [29] and self-efficacy regarding PA behavior change [35,36,39]. Health-related quality of life (SF-12) [40], symptoms of depression (CES-D) [41] and social support will be assessed. Sections of the questionnaire (variables that could change between measurements), will be applied at the study’s three measurement points, to both the control and intervention groups.

### Data management

All the information provided by participants will be supervised by the research team, and also by independent researchers. Questionnaires and clinical test results will be directly captured in a customized Microsoft Access database at the time of the interview with the participant and at the delivery of clinical test results to the patient. Information from other sources (daily PA, attendance record) will also be captured in the customized Microsoft Access database.

### Sample size

On average, 17% of hypertensive patients at the MSSI meet PA recommendations. (Gallegos-Carrillo K, Salmeron J, Duran-Arenas L: Physical activity and diet counseling to hypertensive patients among Mexican Primary care physicians/forthcoming). Our sample size estimation is based on the aim to increase from 17% to 37% the proportion of patients meeting PA standards. To detect a PA difference of 20% in this population, with a power of 90%, and a level of significance of α = 0.05, 68 patients are required in each group (IG and CG). Considering that patients involved in this type of research have high dropout rates [46], to achieve these 68 participants we will increase our initial estimates of sample size by 40%. The study will thus enroll 224 hypertensive patients, 112 each in IG and CG.

### Statistical analysis

Analysis of effectiveness will be carried out according to an intention to treat analysis.

A baseline comparability analysis will be carried out among the IG and CG, in relation to the variables studied during the three measurement points in the study (0, 24 and 32 weeks). To compare means, T-test or analysis of variance will be conducted, while Mann–Whitney tests will be used to compare variables without a normal distribution. Analysis of covariance will be conducted to assess variables with repeated measures along the study.

Longitudinal generalized mixed models will be developed to account for the repeated measurements (0, 24 and 32 weeks) for each patient. These models will be linear for continuous changes in PA (minutes), and logistic for categorical PA variables.

For the primary outcome variable, models will be adjusted for potential confounding variables that may alter the proportion of patients who meet the minimum recommendation for PA: a) geographic accessibility barriers (e.g. distance to SCC); b) financial (e.g. self-report of financial resources to pay for transportation); c) administrative (e.g. SCC schedules not being compatible with patient’s reported schedules); d) social support (e.g. help with transportation to the SCC); e) conditions of patient (for example: greater physical fitness than other hypertensive patients, discomfort or injury discouraging participation in the program, presence of acute or chronic diseases).

In order to minimize regression bias to the estimated mean, baseline values for each dependent variable will be included in each regression model [47].

#### Cost-effectiveness analysis

Information about participants’ use of health care services and program costs will be collected during the study, to be used in subsequent cost-effectiveness analysis in the event that the intervention proves effective.

### Ethical issues

This study and all its procedures were approved by the Scientific Research National Commission and Ethics Committee of the MSSI. It should be noted that participation in the study is voluntary and participants may withdraw at any time without affecting the health care services they receive in any way. The information obtained as part of this study will be strictly confidential, including identifying data (name, phone and address). Personal data will be stored separately to maintain the confidentiality of medical history, questionnaire and clinical test results.

## Discussion

Physical inactivity is highly prevalent worldwide and contributes to the development of the leading non-communicable diseases, a critical health challenge currently faced by lower-middle and upper-middle income countries [7,48]. Now more than ever, these countries require evidence of locally-effective strategies that can help increase activity levels by meeting population-specific needs, which will reduce the impact of non-communicable disease on national health systems.

Community-based PA promotion programs have been developed in Latin American over the past few years. Results of systematic reviews [49,50] show that several countries, including Brazil, have carried out PA promotions in community and school settings with encouraging results. In addition, projects like GUIA [51] (Guide for Useful Interventions in Brazil and Latin America) facilitate PA promotion by helping to translate research findings into practice.

However, integrated community-based and clinical approaches are also needed will be needed to reducing physical inactivity in specific populations [52]. PHC-based strategies have gained acceptance particularly among clinical populations with high levels of non-communicable disease risk factors, because they target a large percentage of the population that actually uses healthcare services. Counseling and referral schemes have been proven to increase the numbers of people meeting PA recommendations [20-23]. However, evidence regarding such programs is concentrated in high-income countries [53].

The present study will explore the effectiveness of PHC-based intervention in a middle-income country setting, assessing a strategy for linking clinical and community health program areas within the same institution and determining the feasibility of this multidisciplinary program’s approach as a potential model to replicate. We will test the effectiveness of a group program for increasing hypertensive patients’ PA engagement, feasible at the IMSS because it contains both medical and physical activity centers, making clinician referral to free PA resources possible.

Evidence from self-reports indicates that referral schemes are effective in increasing PA as measured by self-reports. Yet, information on referral programs’ influence on patient’s PA, measured via objective method is lacking [22,54]. This study will be the first to use direct measurements of PA to show the effects of an exercise-referral scheme in real-world settings. Although we are aware of the limitations posed by the study’s short duration, this does not compromise its utility for physical activity promotion in patients with chronic conditions.

If this intervention is found to be effective, the information could useful to optimize resources. For example, to design programs according to different stages of change, proposing differentiated actions for patients in each one of the stages, as stated in the transtheoretical model [29]. Namely, carry out actions to encourage the willingness to change for patients in pre-contemplation stages or to strengthen their physically active behavior for patients in maintenance stage.

The study population is sharing characteristics such as initially sedentary lifestyle, willingness to engage in PA, and recent hypertension diagnosis. Sharing these traits could encourage the group to develop a sense of community which would promote self-efficacy and social support [55], a fundamental feature of successful behavior change according to social cognitive theory [35,36].

To ensure the quality of this study, the clinical trial guidelines developed by the CONSORT group will be followed [56]. We will be able to identify the number of patients at the three measurement phases of the study and conduct the analysis according to intention to treat.

Finally, this assessment of effectiveness in the real-life conditions of the IMSS health and social services facilities, will serve as a baseline for these types of intervention studies in the context of Latin America, where the ability to test the feasibility, adherence and sustainability of an exercise-referral scheme has not been explored.

While this study is an effort to try to improve participants’ health and the quality of life through PA promotion, we are aware that this kind of initiative does not address the root causes of physical inactivity. This problem will persist unless both PA behaviors and the causes of inactivity change, a situation which requires approaches focused not only on individual behavior, but also on complex population-level systems and interactions [57].

## Abbreviations

MSSI: Mexican Social Security Institute; PA: Physical activity; SCC: Social Security Centers; PHCU: Primary health care unit; HTN: Hypertension; NCDs: Non-communicable Diseases; CHD: Coronary heart disease; IG: Intervention group; CG: Control group; IPAQ: International physical activity questionnaire; ACSM: American College of Sports Medicine; AHA: American Heart Association; BMI: Body mass index; JNC: Joint National Committee; MHR: Maximum Heart Rate; CES-D: Center for Epidemiological Studies-Depression Scale; SF-12: Short-form 12.

## Competing interests

The authors declare that they have no competing interests.

## Authors’ contributions

All authors contributed to the study design and development the protocol. KG: is the principal investigator of the study, conceived and carried out the study design, MCGP, FL, JS, NS are co-investigators and GV the clinical advisor. KG and FL drafted the paper and MCGP, JS and NS revised the manuscript and contributed to subsequent drafts. All authors read and approved the final manuscript.

## Pre-publication history

The pre-publication history for this paper can be accessed here:

http://www.biomedcentral.com/1471-2458/14/706/prepub
